# Persistent parvovirus B19 viremia with chronic arthralgia treated with ascorbic acid: a case report

**DOI:** 10.1186/1752-1947-9-1

**Published:** 2015-01-05

**Authors:** Aloys Lallement, Christine Zandotti, Philippe Brouqui

**Affiliations:** 1 Méditerranée Infection, AP-HM, MIT Nord, Pôle maladies infectieuses, Institut Hospitalo-Universitaire, Chemin des Bourrely, 13915 Marseille, CEDEX 20, France; 2 Méditerranée Infection, AP-HM, Fédération de bactério-virologie-hygiène, CHU Timone, Institut Hospitalo-Universitaire, 264 rue Saint-Pierre, 13385 Marseille, CEDEX 5, France

**Keywords:** Ascorbic acid, Parvovirus B19, Arthralgia, Chronic

## Abstract

**Introduction:**

According to some studies, ascorbic acid possesses antiviral properties. These studies were mainly focused on the common cold, with very few focusing on other viral infections.

**Case presentation:**

We report the case of a 54-year-old Caucasian woman with chronic arthralgia due to persistent parvovirus B19 viremia. High doses of ascorbic acid treatment were initiated due to the failure of conventional analgesic therapy. Clinical benefit was observed with a simultaneous loss of biological parvovirus B19 viremia.

**Conclusions:**

This observation shows a potential benefit of the use of ascorbic acid against parvovirus B19 infections, even if this case is not sufficient to draw any definite conclusions.

## Introduction

Most immunocompetent people with detectable specific human parvovirus B19 (pvB19) antibodies do not recall ever having had any specific symptoms. Arthritis and arthralgia during acute pvB19 infection are more commonly observed in adults rather than children. The symptoms typically subside in weeks, although the arthropathy may persist for months in 20% of affected women [1]. Therapeutic options for pvB19 chronic arthralgia are limited. Since it was isolated in the 1930s, ascorbic acid has been proposed for treating viral infections. It became particularly popular in the 1970s when Nobel laureate Linus Pauling’s trials concluded that ascorbic acid could prevent and alleviate the common cold, but these results are still debated [2]. The safety of high doses of ascorbic acid is now well proved, especially by recent work on cancer treatment with high-dose injections over a medium-term duration [3]. The efficacy is fickle for the common cold, probably because high doses are necessary [2]. However, high doses of ascorbic acid seem effective against infectious mononucleosis and shingles [4, 5].

## Case presentation

We report the case of a 54-year-old Caucasian woman with no relevant past medical history who presented at an outpatient clinic with unexplained isolated joint pain that had been ongoing for six months. No arthritis, rash, fever or anemia symptoms had been reported at the beginning or during the course of disease. During her physical examination, wrist, elbow, shoulder and knee pain was rated by a visual analog scale (VAS) at 60 out of 100. An initial five-day course of corticosteroid therapy (prednisone, 20 milligrams per day) was initiated by her general practitioner with clinical improvement, but relapse was observed at the end of treatment. A dose of acetaminophen (four to six grams per day) was then prescribed for three weeks, unsuccessfully (VAS=50 out of 100). Finally, a non-steroidal-anti-inflammatory drug ketoprofen was given at 200 milligrams per day for four months, with a moderate efficacy (VAS=30 out of 100) before she was referred to us for medical advice.

Among the tests performed for etiological investigations, high levels of pvB19 immunoglobulin G (IgG) and immunoglobulin M (IgM) were found in her serum and her PCR was positive for six months after the onset of symptoms. Her pvB19 blood serology and PCR were still positive at 11 and 13 months after the onset of symptoms (see Table 1). Her rheumatoid factor, antinuclear, anti-transglutaminase and anti-endomysium immunoglobulin A (IgA) antibodies, as well as anti-gliadin IgG and IgA levels tested at months 11 and 13 were all normal and non-contributive.

**Table 1:** **Patient biological and clinical evolution during medical follow-up**

Date	20 August 2013	22 October 2013	11 November 2013	31 December 2013 ^a^	21 January 2014	30 January 2014 ^b^	06 March 2014
Month after symptoms onset	6	8	10	10	11	11	13
pvB19-PCR^c^	+	+	+	+	-	-	-
pvB19-PCR^d^ CT	32	34	38	39	-	-	-
pvB19-serology IgM^e^	+	+	+	+	+	+	+
pvB19-IgM positivity ratio	6.1	6	6.77	6.7	6.5	NA	5.9
pvB19-serology IgG^f^	+	+	+	+	+	+	+
pvB19-IgG positivity ratio	5.3	4.6	4.4	3.4	3.4	NA	3.8
Analgesic	Ketoprofen	Ketoprofen	Ketoprofen	Ketoprofen	None	None	None
Visual analog scale (/100)	30	30	30	30	5	40	10

All biological analyses were performed on serum (defibrinated plasma).

^a^First ascorbic acid treatment.

^b^Second ascorbic acid treatment.

^c^Parvovirus B 19 polymerase chain reaction.

^d^Parvovirus B 19 polymerase chain reaction cycle threshold.

^e^Immunoglobulin M.

^f^Immunoglubulin G.

NA means NOT Available.

Because of persistent circulating pvB19-DNA and poor treatment response, we decided to stop the treatment and to begin oral acid ascorbic at 10 grams per day for 10 days, as suggested in other viral illnesses. At five days after the initiation of treatment, she described an important decrease in symptoms (VAS=5). At three weeks after treatment, a pvB19-PCR analysis on her serum was negative for NS1 protein encoding gene, while her serology was still positive for IgG and IgM. A relapse occurred three weeks after the end of treatment, but she fully recovered after a second administration of the same dose for three complementary weeks. At the time of this report, she remains completely recovered.

## Discussion

Few prior studies have reported on the treatment of persistent isolated joint pains due to pvB19 infection. In cases of acute arthropathy, non-steroidal anti-inflammatory drugs may exert beneficial effects and are often recommended in reviews [6–8], but no therapeutic trial has explored the specific association of pvB19 chronic arthropathy and non-steroidal anti-inflammatory drug efficiency. Intravenous immunoglobulin therapy has been tested in severe arthritis associated with pvB19 infection, with good results in non-isolated arthropathy [9–14], but non-contributive results in the case of isolated severe chronic arthropathy [15–17]. While chloroquine has a wide antiviral activity, *in vitro* experiments on 4-aminoquinoline drugs and their metabolites suggest that they exacerbate pvB19-associated anemia by promoting replication [18]. While there is no data *in vitro* or *in vivo* in the literature on the efficacy of ascorbic acid on pvB19, the safety of high-dose ascorbic acid relative to the potential benefit justifies the prescription of this therapy.

## Conclusions

Whilst we are aware that the negativity of the pvB19-PCR analysis and the clinical improvement of our patient may be independent and coincidental, we suggest a possible efficacy when high doses of ascorbic acid are used for treating persistent pvB19 infections. Considering the limited therapeutic options for chronic pvB19-associated joint pain and the lack of side effects associated with ascorbic acid treatment, we suggest using the latter when other therapies have failed.

## Consent

Written informed consent was obtained from the patient for publication of this case report and any accompanying images. A copy of the written consent is available for review by the Editor-in-Chief of this journal.

## Abbreviations

DNA: deoxyribonucleic acid
IgA: Immunoglubulin A
IgG: Immunoglubulin G
IgM: Immunoglobulin M
pvB19: parvovirus B19
PCR: polymerase chain reaction
VAS: visual analog scale.

## References

[1] Woolf AD, Campion GV, Chishick A, Wise S, Cohen BJ, Klouda PT, Caul O, Dieppe PA: **Clinical manifestations of human parvovirus B19 in adults.** *Arch Intern Med* 1989, **149:**1153–1156. 10.1001/archinte.1989.00390050111022 2541666

[2] Hemilä H, Chalker E: **Vitamin C for preventing and treating the common cold.** *Cochrane Database Syst Rev* 2013., **1:** CD000980 10.1002/14651858.CD000980.pub4 PMC8078152 23440782

[3] Stephenson C, Levin D, Spector T, Lis G: **Phase 1 clinical trial to evaluate the safety, tolerability and pharmacokinetics of high-dose intravenous ascorbic acid in patients with advanced cancer.** *Cancer Chemother Pharmacol* 2013, **72:**139–146. 10.1007/s00280-013-2179-9 10.1007/s00280-013-2179-9 PMC3691494 23670640

[4] Mikirova N, Hunninghake R: **Effect of high dose vitamin C on Epstein-Barr viral infection.** *Med Sci Monit* 2014, **20:**725–732. 10.12659/MSM.890423 PMC4015650 24793092

[5] Schencking M, Vollbracht C, Weiss G, Lebert J, Biller A, Goyvaerts B, Kraft K: **Intravenous vitamin C in the treatment of shingles: results of a multicenter prospective cohirt study.** *Med Sci Monit* 2012, **18:**215–224. 10.12659/MSM.882621 PMC3560828 22460093

[6] Franssila R, Hedman K: **Infection and musculoskeletal conditions: viral causes of arthritis.** *Best Pract Res Clin Rheumatol* 2006, **20:**1139–1157. 10.1016/j.berh.2006.08.007 10.1016/j.berh.2006.08.007 17127201

[7] Heegaard ED, Brown KE: **Human parvovirus B19.** *Clin Microbiol Rev* 2002, **15:**485–505. 10.1128/CMR.15.3.485-505.2002 10.1128/CMR.15.3.485-505.2002 PMC118081 12097253

[8] Gallinella G: **Parvovirus B19 achievements and challenges.** *ISRN Virology* 2013, **2013:**898730.

[9] Bozzola E, Krzysztofiak A, Cortis E: **Neurological impairment and arthritis in an immunocompetent child with human parvovirus B19 chronic infection.** *Infez Med* 2010, **18:**187–190. 20956876

[10] Jacobson SK, Daly JS, Thorne GM, McIntosh K: **Chronic parvovirus B19 infection resulting in chronic fatigue syndrome: case history and review.** *Clin Infect Dis* 1997, **24:**1048–1051. 10.1086/513627 10.1086/513627 9195056

[11] Frickhofen N, Abkowitz JL, Safford M, Berry JM, Antunez-de-Mayolo J, Astrow A, Cohen R, Halperin I, King L, Mintzer D, Cohen B, Young NS: **Persistent B19 parvovirus infection in patients infected with human immunodeficiency virus type 1 (HIV-1): a treatable cause of anemia in AIDS.** *Ann Intern Med* 1990, **113:**926–933. 10.7326/0003-4819-113-12-926 10.7326/0003-4819-113-12-926 2173460

[12] Frickhofen N, Chen ZJ, Young NS, Cohen BJ, Heimpel H, Abkowitz JL: **Parvovirus B19 as a cause of acquired chronic pure red cell aplasia.** *BR J Haematol* 1994, **87:**818–824. 10.1111/j.1365-2141.1994.tb06743.x 10.1111/j.1365-2141.1994.tb06743.x 7986722

[13] Koduri PR, Kumapley R, Valladares J, Teter C: **Chronic pure red cell aplasia caused by parvovirus B19 in AIDS: use of intravenous immunoglobulin--a report of eight patients.** *Am J Hematol* 1999, **61:**16–20. 10.1002/(SICI)1096-8652(199905)61:1<16::AID-AJH4>3.0.CO;2-Y 10.1002/(sici)1096-8652(199905)61:1<16::aid-ajh4>3.0.co;2-y 10331506

[14] Kurtzman GJ, Cohen BJ, Field AM, Oseas R, Blease RM, Young NS: **Immune response to B19 parvovirus and an antibody defect in persistent viral infection.** *J Clin Invest* 1989, **84:**1114–1123. 10.1172/JCI114274 10.1172/JCI114274 PMC329767 2551923

[15] Stahl HD, Pfeiffer R, Emmrich F: **Intravenous treatment with immunoglobulins may improve chronic undifferentiated mono- and oligoarthritis.** *Clin Exp Rheumatol* 2000, **18:**515–517. 10949732

[16] Ogawa E, Otaguro S, Murata M, Kainuma M, Sawayama Y, Furusyo N, Hayashi J: **Intravenous immunoglobulin therapy for severe arthritis associated with human parvovirus B19 infection.** *J Infect Chemother* 2008, **14:**377–382. 10.1007/s10156-008-0636-X 10.1007/s10156-008-0636-x 18936893

[17] Saag KG, True CA, Naides SJ: **Intravenous immunoglobulin treatment of chronic parvovirus B19 arthropathy.** *Arthritis Rheum* 1993,**36**(Suppl)**:**S67.

[18] Bönsch C, Kempf C, Mueller I, Manning L, Laman M, Davis TM, Ros C: **Chloroquine and its derivatives exacerbate B19V-associated anemia by promoting viral replication.** *PLoS Negl Trop Dis* 2010, **4:**e669. 10.1371/journal.pntd.0000669 10.1371/journal.pntd.0000669 PMC2860510 20436917

